# Feather RNA: A Non-Invasive Approach for Transcriptomic Profiling in Live Chickens

**DOI:** 10.3390/vetsci13070653

**Published:** 2026-07-05

**Authors:** Nadia Stoppani, Federica Raspa, Edoardo Fiorilla, Sandra Maione, Achille Schiavone, Cecilia Mugnai, Dominga Soglia

**Affiliations:** Dipartimento di Scienze Veterinarie, Università degli Studi di Torino, Largo Paolo Braccini 2, Grugliasco, 10095 Turin, Italy; nadia.stoppani@unito.it (N.S.); federica.raspa@unito.it (F.R.); edoardo.fiorilla@unito.it (E.F.); sandra.maione@unito.it (S.M.); achille.schiavone@unito.it (A.S.); cecilia.mugnai@unito.it (C.M.)

**Keywords:** feathers, gene expression, chicken, candidate gene

## Abstract

In this study, an exploratory transcriptomic investigation was conducted to evaluate the feasibility of using feather transcriptomics to detect sex differences and gene responses to physiological changes in chickens. Feathers represent a promising non-invasive biological source of RNA, as the feather pulp of growing feathers contains living cells capable of active transcription. Growing feathers were collected from 150-day-old male and female chickens (Bionda Piemontese, a slow-growing breed) raised under a free-range system and fed two finisher diets differing in lipid content: low-lipid (LL, ether extract 3.6%) and high-lipid (HL, ether extract 9.3%) diets. RNA was extracted from feather pulp, and 12 pools were subjected to whole RNA-Seq analysis. The study was designed as 2 × 2 factorial experiments investigating the effects of diet and sex on gene expression. A total of 17,360 transcripts were detected and used for downstream analyses. Differential gene expression and functional enrichment analyses were performed. The main effects of diet and sex were estimated with an additive design using the DEseq2 package, while for the sex-specific diet analyses, subgroup comparisons were conducted on the RaNA-Seq platform. The analysis of the main effect of diet reveals that three genes associated with ether lipid metabolism (PLA2G10, PLA2G4F, and ENPP6) were upregulated in chickens fed the HL diet. In roosters, HL feeding significantly altered the expression of APOA1 and SLC27A4, suggesting an effect on lipid transport and metabolic regulation within the PPAR signaling pathway. In contrast, hens showed differential expression primarily in pathways related to apelin signaling, extracellular matrix remodeling, and cardiovascular function, rather than classical lipid metabolism pathways; additionally, gene set enrichment analysis indicated a limited enrichment of linoleic acid metabolism, suggesting secondary involvement of lipid metabolic processes. These findings are consistent with those in the literature reporting sex-related differences between males and females. The results further suggest that transcriptomic responses to dietary lipid supplementation can be investigated through the expression of selected candidate genes in feather pulp. Among the genes identified, PLA2G10, PLA2G4F, ENPP6, APOA1, and SLC27A4 emerged as potential molecular markers associated with dietary treatment, and the importance of sex-dependent transcriptional responses was highlighted. In conclusion, this study demonstrates the potential of feather pulp as a viable source of RNA for transcriptomic analyses in live chickens, providing a minimally invasive alternative to conventional tissue sampling. These preliminary results also support the hypothesis that feathers represent a practical and ethically favorable tissue for future nutrigenomic and genetic improvement studies, ultimately supporting more sustainable poultry production.

## Simple Summary

In this study, we suggest the potential application of feathers as a non-invasive tissue to study the genetic response to physiological stimuli, as an effect of diet, in live chickens. The results suggest that feather transcriptomics can capture nutrigenomic responses. Therefore, feather pulp is a promising exploratory matrix for detecting group-level transcriptional trends. While some candidate genes were identified, further studies are still needed.

## 1. Introduction

Feathers represent a non-invasive biological sample suitable for genetic analysis in birds, as they can be collected directly from live animals without the need for euthanasia [1]. Structurally, feathers are characterized by a calamus and a rachis, which provide support to the feather. During feather growth, feather follicles develop: the dermal papilla grows upward, into the calamus, to form the pulp, and endothelial cells give rise to capillary vessels, which transport nutrients to the various parts of the feather. The feather follicle is a tissue characterized by the presence of stem cells, which have strong potential for multidirectional proliferation, division, and differentiation. Stem cells can sense changes in microenvironment signals and modulate follicle growth, which is critical for the regeneration and growth of feathers [2,3,4]. Therefore, the calamus represents the only part that contains living cells (feather pulp) during feather growth, making it useful for genetic analysis. GWASs (Genome-Wide Association Studies) on poultry feather follicle development primarily focus on identifying genetic markers associated with feather-related traits, while transcriptomics studies focus on gene expression changes within follicle cells and their effect on feather formation, development, and growth. Gene expression in feathers is primarily associated with regenerative and physiological needs, trauma response, and bird plumage pigmentation [5,6,7,8].

Beyond their role in pigmentation and regenerative processes, gene expression in feathers may also reflect systemic metabolic pathways. Feathers are dynamic biological structures that, during their growth phase, integrate signals arising from multiple physiological and environmental dimensions. Beyond physiological processes, a growing body of evidence indicates that feathers also respond to environmental and behavioral stimuli; variations in habitat conditions, including diet composition, can modulate feather follicle stem cells [2,3,9].

Nutrients are essential for the development of feather follicles, as well as the feathers themselves: experimental studies demonstrate that changes in diet quality and food availability directly influence feather growth, supporting its strong relationship with an individual’s energy status, highlighting the utility of feathers as indicators of nutritional stress [2].

Collectively, these findings support the concept that feathers are not merely passive structures but rather biological archives that integrate physiological, environmental, and nutritional information over time. This integrative property makes them a powerful tool for assessing animal welfare and investigating diet interactions.

In addition, feathers can also be exploited for gene expression studies, as the living cells present in the calamus provide RNA suitable for transcriptomic analyses. The tissue within the follicle of a new feather can be extracted and analyzed. Because feathers contain living cells capable of active transcription, as well as blood, the transcriptome of their pulp could provide information on their metabolic functions. These living cells are particularly accessible during plumage that occurs while molting or following damage [4].

Traditionally, RNA expression related to the effect of diet in chickens has been studied in their livers [10,11], abdominal fat [12], or breast muscles [10,13,14], all of which require euthanizing the birds. Consistent with these findings, most transcriptomic variations are reportedly found in blood rather than in the liver or adipose tissue, and clear sexual dimorphism has been highlighted [10,11,15,16].

In this study, we aimed to evaluate the potential of feathers as a non-invasive tissue for assessing genetic responses to physiological stimuli, such as dietary variation, in live male and female chickens.

## 2. Materials and Methods

### 2.1. Animals and Experimental Diets

The trial was conducted at the poultry facility of the University of Turin. Details of the animal rearing methods are reported in [15]. Briefly, a total of 60 chickens (30 males and 30 females) of the Bionda Piemontese breed, a slow-growing breed native to North-west Italy, were used for this trial. All chickens were raised in a free-range production system, where they were subjected to natural environmental conditions, including natural temperature conditions and photoperiods, and had free access to outdoor areas. Throughout the starter and growing phases, all birds received the same diet.

During the finisher phase (from 120 to 150 days of age), males and females were allocated to two dietary treatments: a control group receiving a standard low-lipid diet (LL, ether extract, EE = 3.6%) and a high-lipid diet (HL, EE = 9.3%) prepared by adding 6% of palm kernel. The HL diet showed a 61.6% increase in lipid content compared to the control diet. Details of each feed formulation are provided in Table 1.

**Figure 1:**
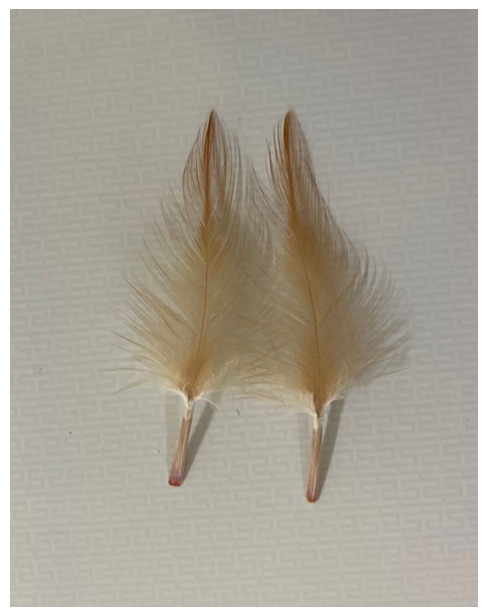
Example of growing feathers. The feathers are characterized by the presence of pulp in the calamus.

Growing feathers were collected from body areas showing active regrowth near the neck and along the dorsal tract in 60 BP chickens (15 males and 15 females per diet) at 150 days old. All collected feathers were characterized by the presence of pulp in the calamus (Figure 1). The feathers were then put in RNAlater Stabilization Reagent (QIAGEN, Hilden, Germania) and stored at −80 °C until extraction.

### 2.2. Experimental Design, Sample Pooling, and RNA Sequencing

Total RNA was extracted from feathers (specifically from the pulp) using the FastGene^®^ RNA Premium Kit (Nippon Genetics Europe, Düren, Germany). For each sample, approximately 2 mg of tissue was collected from the base of the feather calamus containing pulp. RNA quantity was assessed using RNA Broad-Range Assay Kit on Qubit 3^®^ Fluorometer (Invitrogen, Thermo Fisher Scientific, Waltham, MA, USA), and RNA integrity number (RIN) was evaluated using the Agilent RNA 6000 Nano Kit on an Agilent 2100 Bioanalyzer (Agilent Technologies, Santa Clara, CA, USA).

Only samples with a minimum concentration of 100 ng/µL and an RNA Integrity Number (RIN) ≥ 7 were considered for subsequent analyses.

A total of 24 RNA samples were selected for equal distribution among the four experimental groups (*n* = 6/group): males fed the HL diet, males fed the LL diet, females fed the HL diet, and females fed the LL diet. Within each experimental group, two individual RNAs were pooled at the same RNA concentration and in equal proportions to generate three independent pools per group, resulting in a total of 12 pooled libraries for sequencing.

As a result, each library represents a pooled sample rather than an individual organism. Consequently, the experimental unit for statistical analysis was defined as the pooled sample.

An aliquot of each pool was sent to AZENTA (Azenta Life Sciences, Chelmsford, MA, USA) for transcriptomic profiling. Sequencing data were provided in FASTQ format.

### 2.3. RaNA-Seq: Differential Expression and Functional Enrichment Analysis

The RaNA-Seq open bioinformatics tool (https://ranaseq.eu/ accessed on 1 July 2026) was used to analyze RNA-Seq data. It quantifies FASTQ files based on the alignment of the reference genome, calculates the quality of reads (Salmon software 2.0), performs a normalization test (TPMs, Transcript Per Millions), runs a differential expression analysis (DESeq2), and interprets the results based on a functional analysis [17]. The RaNa-Seq process is well explained in [17]. Briefly, the reference genome used to assemble the reads was Gallus gallus-5.0. Once the files in FASTQ format were uploaded to the RaNA-Seq software, the reads were filtered based on their quality and processed with Salmon software to quantify the reads for each gene. Only uniquely mapped reads were retained for downstream analyses. Gene expression levels were normalized as TPMs [18]. Differential gene expression analysis was performed by the RaNA-seq tool using the DESeq2 package, applying a statistical model based on negative binomial distribution. *p*-value was corrected for multiple testing using the Benjamini–Hochberg procedure. Genes were considered differentially expressed when the adjusted *p*-value (padj) was <0.05. Results reports contain the volcano plot figures which could help to explore and interpret results. Volcano plot shows the log2 scaled fold change (*x*-axis) and the minus log10 *p*-value (*y*-axis) of each gene in the differential expression analysis. Genes with a significant (padj < 0.05) expression change are highlighted as red dots. It is important to note that the software allowed only pairwise comparisons between experimental conditions. Therefore, following the flow chart in [18], a differential expression analysis was performed to compare the effects of each diet for each sex. Functional enrichment analysis and Gene Set Enrichment Analysis (GSEA) were performed to investigate the biological processes and pathways associated with gene expression changes. The tool uses the GOseq R package for this analysis [19]. By default, the software uses the Kyoto Encyclopedia of Genes and Genomes (KEGG) and WikiPathways databases for pathway identification. The results from the functional enrichment analysis were selected based on a *p*-value threshold (*p* < 0.05). In contrast, for GSEA, both padj and the normalized enrichment score (NES) were taken into account, providing a more rigorous assessment of statistical significance. NES values reflect both the direction and magnitude of enrichment, with positive scores indicating enrichment at the top of the ranked list and negative scores indicating enrichment at the bottom. Pathways with |NES| ≥ 2 were considered significant enrichment signals. However, since no multiple-testing correction was available, these findings should be considered exploratory and interpreted with caution. Therefore, the analysis was restricted to individual pairwise contrasts, limiting the ability to assess the combined effects of multiple experimental factors within a single statistical framework.

To overcome this limitation, differential gene expression analysis was performed in R using DESeq2, outside the RaNA-seq tool.

### 2.4. R-Package: Differential Expression and Functional Enrichment Analysis

A differential expression analysis was modeled using a negative binomial generalized linear model implemented in DESeq2. Low-count genes were filtered prior to differential expression analysis by retaining only genes with a total read count greater than 10 across all samples.

Initially, a factorial model incorporating the sex × diet interaction term was explored. While no interaction effects were detected, the low resolution inherent in aggregate sampling rendered these findings insufficiently robust for reliable biological interpretation; in fact, the pooled nature of the samples limits the ability to reliably estimate within-group biological variability required for robust interaction inference. Therefore, they were not considered further. For this reason, an additive design was used to estimate the main effects:

design = ~ sex + diet

Differential expression was assessed using Wald tests as implemented in DESeq2. The following comparisons were evaluated:

Main effect of diet (HL vs. LL diet, averaged across sexes)

Main effect of sex (male vs. female, averaged across diets)

*p*-value was corrected for multiple testing using the Benjamini–Hochberg procedure. Genes were considered differentially expressed when padj was <0.05. In addition, the log2 fold change (log_2_FC) was used to assess the magnitude and direction of gene expression changes, allowing for the identification of upregulated (log_2_FC > 1) and downregulated (log_2_FC < −1) genes.

Functional enrichment analysis and Gene Set Enrichment Analysis (GSEA) were performed in R using differential expression results obtained from the additive model including sex and diet as main effects. Principal Component Analysis (PCA) was performed on the variance-stabilized gene expression data obtained using the variance stabilizing transformation (VST) implemented in the DESeq2 package in R. PCA was used to assess sample clustering, identify potential outliers, and evaluate the overall variability among samples. The first two principal components (PC1 and PC2) were visualized using the plotPCA function from DESeq2. Hierarchical clustering heatmap of whole-transcriptome RNA-seq samples based on pairwise sample similarity (using VST) was performed with pheatmap function in R.

Comparisons were visualized using the Enhanced Volcano package in R: red dots represent significant upregulated DEGs (padj < 0.05; Log_2_FC > 1) and blue dots significant downregulated DEGs (padj < 0.05; Log_2_FC < −1). Genes with statistical significance but which did not surpass any fold-change thresholds are displayed as dark gray dots (padj < 0.05; −1 ≤ Log_2_FC ≤ 1), and non-significant genes (padj ≥ 0.05) are shown as light gray dots. The x-axis shows the Log_2_FC of each gene; the y-axis shows the minus log10 *p*-value calculated using the selected analysis method for each gene.

## 3. Results

### 3.1. RNA Extraction from Feathers

A mean RNA extraction yield of 456 ± 234 ng/μL was obtained from the feather samples. In total, about 2.5 × 10^7^ reads were sequenced from each library, and 92% were aligned with the Gallus gallus-5.0 genome. Quality control results (Supplementary Materials, Figure S1) showed that the expression was distributed homogeneously in all samples, meaning that no sequencing or sample collection problems occurred. The resulting PCA plots and heatmaps are provided in the Supplementary Materials (Figures S2 and S3). In total, 17,360 transcripts were detected across all samples.

### 3.2. Main Effect of Diet

**Figure 2:**
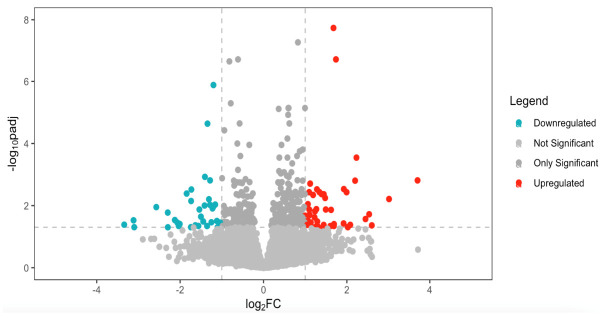
Volcano plot of significantly differentially expressed genes (DEGs) between the two diets.

Differential expression analysis (additive design) between the two dietary treatments detected 387 annotated significant genes (padj < 0.05). Figure 2 shows a Volcano plot of the obtained differentially expressed genes using the additive design in R software (version 4.3.0).

In particular, three significant genes associated with ether lipid metabolism were identified: PLA2G10, ENPP6, and PLA2G4F. All genes showed a trend of upregulation in the HL diet compared to in the LL diet (padj = 0.04, 0.02, and 0.03; log_2_FC = 0.73, 0.88, and 0.65, respectively). The main altered pathways are shown in Supplementary Materials Figure S4.

**Figure 3:**
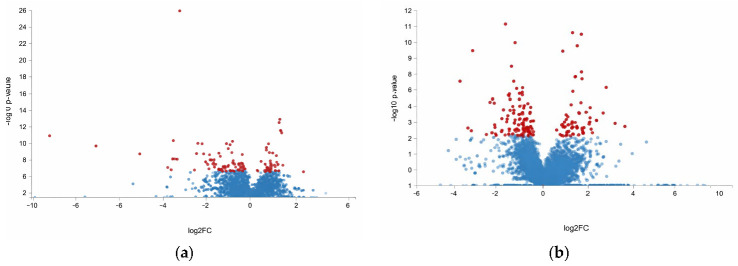
Volcano plot of significantly differentially expressed genes (DEGs) between the two diets in roosters (**a**) and hens (**b**). Red dots represent significantly different expressed gene (padj < 0.05), while in blue are represented the not significant ones (padj ≥ 0.05).

In the subgroup comparisons using RaNA-seq tool, a separate sex-stratified RNA-seq analysis identified 118 in roosters and 153 DEGs in hens (Figure 3).

In roosters, RaNA-seq functional enrichment analysis highlighted the involvement of cell cycle, AGE-RAGE signaling, insulin signaling, p53, and PPAR pathways. Consistently, GSEA confirmed the activation of proliferative programs (cell cycle) and further indicated modulation of the PPAR pathway. Interestingly, within PPAR pathways, involved in energy and lipid metabolism, APOA1 and SLC27A4 were significantly upregulated in rooster fed the HL diet (padj = 0.01, and 0.02, respectively), with a markedly stronger effect for APOA1 (log_2_FC = 1.69) compared to SLC27A4 (log_2_FC = 0.34). Furthermore, GSEA revealed a coordinated downregulation of metabolic pathways (NES ≤ −2), including oxidative phosphorylation, ribosome biogenesis, and proteasome activity. This reduction in metabolic and mitochondrial activity is characteristic of actively proliferating or transformed cells.

In females, RaNA-seq functional enrichment analysis revealed the involvement of the apelin signaling pathway, extracellular matrix (ECM) remodeling mediated by metalloproteinases (MMPs), and cardiac muscle contraction. No pathways directly related to lipid metabolism were identified. GSEA additionally revealed a significant enrichment of the linoleic acid metabolism pathway (padj = 0.02; NES = −2.00), suggesting the regulation of lipid metabolic processes.

**Figure 4:**
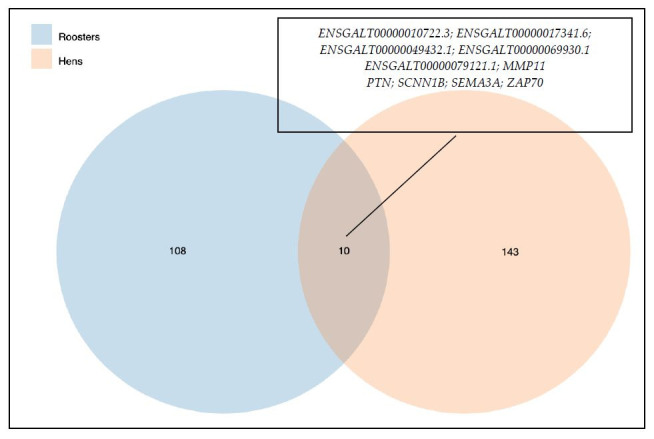
Venn diagram of significantly differently expressed genes (DEGs) between the two dietary treatments in roosters and hens: number of DEGs (padj < 0.05) between high-lipid (HL) and low-lipid (LL) diets in roosters (in light blue) and Hens (in light orange). The overlapping region highlights 10 genes showing diet-dependent expression changes independent of sex.

The Venn diagram in Figure 4 illustrates and compares the number of differentially expressed genes (DEGs) identified in the LL and HL dietary treatments for both males and females. Overlapping areas indicate genes commonly differentially expressed between the two groups: a total of 10 DEGs were shared between roosters and hens. These include genes involved in extracellular matrix remodeling (MMP11), neural development (SEMA3A and PTN), immune signaling (ZAP70), and ion transport (SCNN1B). The remaining five DEGs were annotated with Ensembl transcript IDs, indicating transcripts not yet assigned to official gene symbols in the current Gallus gallus annotation. These transcripts have been reported as novel in the Red Junglefowl, a local chicken breed from India, although further characterization studies are still lacking. Most gene expression differences emerged when sex was analyzed within each dietary condition, suggesting sex-specific responses to diet.

### 3.3. Main Effect of Sex

**Figure 5:**
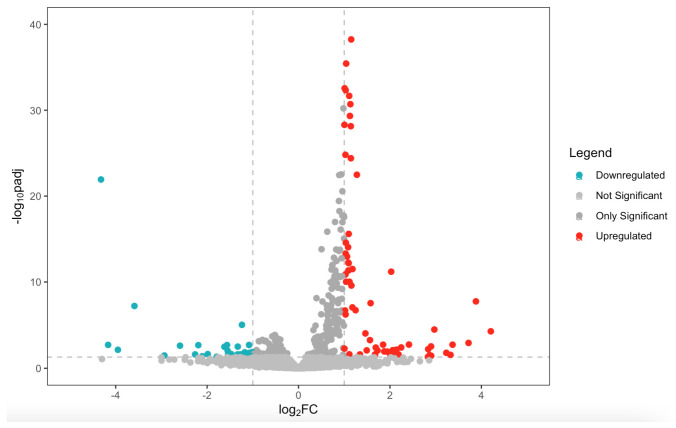
Volcano plot of significantly differentially expressed genes (DEGs) between the two sexes.

The effect of sex, evaluated through differential expression analysis using an additive design, revealed a total of 346 significant annotated genes between males and females (padj < 0.05). The volcano plot in Figure 5 illustrates these differentially expressed genes using R software. The main altered pathways are shown in the Supplementary Materials, Figure S5.

## 4. Discussion

This study was designed as an exploratory investigation aimed at characterizing feathers as a novel biological matrix in transcriptomic analysis.

The results suggest the potential future application of feathers as a non-invasive tissue for investigating metabolic changes associated with physiological or environmental perturbation in live chickens. In fact, feather pulp appears to be a promising tissue for RNA extraction and for detecting both broad and nuanced gene expression patterns associated with dietary lipid exposure. In particular, this study analyzed feather-derived transcriptomes from chickens exposed to high-lipid (HL) or low-lipid (LL) diets.

The use of pooled RNA samples in the present study was primarily motivated by the exploratory nature of the experiment and the limited economic resources available. Since this work aimed to provide a preliminary evaluation of feather pulp as a novel and minimally invasive matrix for transcriptomic analyses in chickens, pooling represented a practical and cost-effective strategy to assess the feasibility of detecting biologically meaningful transcriptional differences associated with sex and dietary treatments. In pilot RNA-seq studies, especially when testing unconventional biological matrices, pooling can help generate an overall representation of the transcriptional profile while reducing library preparation and sequencing costs. This approach was therefore considered appropriate for obtaining an initial overview of gene expression patterns and identifying potential pathways and candidate genes responsive to physiological and nutritional differences. Another advantage of pooling is the reduction in interindividual variability, which can facilitate the detection of common transcriptional signals within experimental groups. In the present study, this strategy enabled the identification of sex differences in diet responses, supporting the potential applicability of feather transcriptomics in nutritional and physiological investigations in chickens. Importantly, the observed sex responses are consistent with those in the existing literature: marked metabolic and transcriptional differences exist between males and females. However, some limitations associated with pooling must also be acknowledged. Because pooled samples represent an average biological profile, individual variability could not be evaluated, and the statistical power for detecting individual-specific responses was reduced. In addition, PCA identified one pooled sample that displayed a distinct clustering pattern and could therefore be considered a potential outlier. However, given the limited number of samples available, the pool was retained in the analysis to avoid further reducing statistical power. Consequently, the results should be interpreted as preliminary and hypothesis-generating rather than definitive evidence of differential gene expression. In particular, subtle individual responses to dietary treatments may have been masked by the pooling strategy.

Although the highest proportion of genes was annotated to the Epidermal Growth Factor Re-ceptor (EGFR) signaling pathway, which regulates growth, survival, proliferation, and differentiation [20], the inferential analysis for the main effects of diet showed that an HL diet upregulated PLA2G10, PLA2G4F, and ENPP6, suggesting the potential coordinated modulation of phospholipid-related metabolic processes. PLA2G10 and PLA2G4F are members of the phospholipase A2 (PLA2) family, which plays an essential role in signal transduction, phospholipid remodeling, membrane homeostasis, and energy production for fatty acid β-oxidation in mammals [21]. Specifically, the PLA2G10 gene is involved in the selective hydrolysis of phospholipids and plays a crucial role in fat metabolism and energy utilization in pigs [22], whereas PLA2G4F has been associated with intramuscular fat-related traits in poultry and other avian models [23,24]. ENPP6 encodes a choline-releasing enzyme involved in phospholipid catabolism and choline homeostasis, particularly in hepatic metabolism [25], and has also been suggested to participate in extracellular matrix-related processes under specific physiological contexts [26]. Although intramuscular fat content was not directly assessed in this study, previous results obtained using the same experimental framework indicated reduced thawing loss in males fed the HL diet [15], which may be consistent with changes in muscle composition or membrane-related properties. However, such an association remains speculative in the absence of direct physiological measurements. Overall, the concurrent modulation of ENPP6 and PLA2-related genes may reflect a broader adjustment in phospholipid turnover and choline-related metabolic pathways potentially associated with dietary lipid intake.

In the subgroup comparison analysis, males and females exhibited distinct secondary transcriptional responses.

In males, functional enrichment analysis and GSEA both indicated the involvement of insulin signaling and PPAR pathways, both of which are involved in energy and lipid metabolism [27,28], in response to the HL diet. The concordant results from functional enrichment analysis and GSEA suggest a likely involvement of insulin signaling and PPAR pathways in the male response to the HL diet. In particular, increased expression of genes such as APOA1 and SLC27A4 may suggest a modulation of lipid transport and fatty acid uptake processes. APOA1 has been associated with lipid and cholesterol transport in chickens, although its regulation appears to vary depending on breed, tissue, and production system [29,30,31]. In the present study, its upregulation in HL-fed males may indicate a potential adaptive response to increased dietary lipid availability, although this interpretation remains hypothetical in the absence of direct metabolic measurements. Similarly, SLC27A4, a gene involved in fatty acid transport and activation, has been proposed as a candidate gene for lipid deposition traits in poultry [32]. Its upregulation in HL-fed chickens may reflect an increased capacity for fatty acid uptake and activation in response to the elevated dietary lipid load. However, this response should not be directly interpreted as evidence of increased lipid deposition, as gene expression changes may reflect transient metabolic adjustments rather than final phenotypic outcomes.

In females, functional enrichment analysis showed an over-representation of pathways related to apelin signaling, ECM remodeling mediated by MMPs, and cardiac and vascular function. None of these pathways are directly involved in lipid metabolism. However, GSEA revealed a significant enrichment of the linoleic acid metabolism pathway. In chickens, the apelin signaling pathway is primarily associated with the initiation of sexual maturity [33], ECM remodeling with oviduct development [34], and the cardiac/vascular pathway with heart contraction.

These results, which should be confirmed in a larger study, suggest that females may exhibit a more distributed transcriptional response to dietary lipids, involving reproductive and cardiovascular-related pathways, alongside a weaker but coordinated modulation of lipid metabolism. This highlights the importance of considering sex-specific metabolic and transcriptional programs in nutritional studies.

It is important to note that these findings differ from the hepatic gene expression results previously reported in the same chicken breed, which were based on an analysis using a panel of nine target genes [15]. This apparent discrepancy may be explained by the marked tissue-specific expression patterns of many genes, some of which are highly expressed in the liver but show little or no expression in feather pulp. More generally, these findings highlight the strong tissue dependency of gene expression responses to dietary interventions. Importantly, the present data demonstrate that gene transcripts can be detected and quantified in feather pulp, supporting its use as a non-invasive source of transcriptional information. In addition, the findings indicate that gene transcription in feather pulp can be influenced by dietary regimens. Consequently, the differentially expressed genes identified in this study may represent potential feather pulp-specific candidate genes for detecting diet-induced metabolic changes in live chickens, although their biological relevance and practical applicability require further validation.

Despite the limited sample size and the use of pooled samples, the results are consistent with those of previous studies reporting clear transcriptomic differences between males and females. Although these limitations prevent definitive conclusions, the adopted approach provides preliminary evidence supporting the feasibility of using feather pulp as a transcriptomic matrix and offers valuable insights for future research.

The findings presented here may therefore serve as a foundation for larger-scale studies incorporating individual biological replicates. Such investigations will be essential to validate the molecular signatures identified in this study and to further characterize sex- and diet-related transcriptional responses in this local chicken breed.

Taken together, these results suggest that feathers represent a feasible non-invasive tissue for detecting diet-induced metabolic and sex-specific transcriptional responses. A high-lipid diet appears to induce a steady early response potentially related to phospholipid-remodeling mechanisms across diets, while subgroup comparisons showed different responses of males and females to diet: males showed transcriptional signals consistent with lipid transport and catabolism, whereas females exhibited pathway-level changes associated with reproductive and cardiovascular physiology, along with a minimum effect on the linoleic acid metabolism pathway. These differences may reflect sex-specific regulatory mechanisms, potentially influenced by hormonal regulation, which could contribute to a differential allocation of metabolic resources in response to dietary lipids.

The transcriptomic results should be interpreted as representative of pooled biological samples and therefore reflect the average gene expression profile of each experimental group rather than individual biological variability. Nevertheless, the present study was conceived as an exploratory investigation aimed at evaluating feather pulp as a novel and minimally invasive biological matrix for transcriptomic studies in poultry. Within this proof-of-concept framework, RNA-Seq was employed as a hypothesis-generating approach to identify molecular signatures and biological pathways potentially associated with the experimental conditions. Consequently, no orthogonal validation was performed at this stage. Thus, future studies involving individual samples and larger cohorts are needed to validate the identified transcriptional patterns and further assess the utility of feather pulp for molecular and functional genomic investigations in poultry.

## 5. Conclusions

In conclusion, this study demonstrates the potential of feather pulp as a viable and ethically advantageous source of RNA for transcriptomic analyses in live chickens, providing a minimally invasive alternative to conventional tissue sampling. This approach is particularly valuable for local and endangered breeds, for which limited population size and conservation concerns restrict destructive sampling procedures associated with slaughtering. Consequently, feather pulp represents a promising tool for both conservation research and genetic improvement programs. The results further suggest that transcriptomic responses to dietary lipid supplementation can be investigated through the expression of selected candidate genes in feather pulp. Among the genes identified, PLA2G10, PLA2G4F, ENPP6, APOA1, and SLC27A4 emerged as potential molecular markers associated with dietary treatment, and the importance of sex-dependent transcriptional responses was highlighted. However, these findings should be interpreted with caution due to the use of pooled samples and the lack of independent validation. Therefore, the present study should be regarded as a proof-of-concept providing preliminary evidence rather than definitive conclusions.

Overall, these findings support the use of feather pulp as an exploratory biological transcriptomic matrix and provide a basis for future studies using larger populations, individual-level sampling, and independent validation to develop reliable molecular biomarkers for precision nutrition, breeding, and the conservation of poultry genetic resources. Additionally, it could serve as a matrix for monitoring group-level transcriptional changes in poultry.

## Figures and Tables

**Table 1:** Diet ingredients and chemical composition.

Ingredients (g/kg as Fed)	LL	HL
Corn meal	700	625
Soybean meal	224	239
Palm kernel oil	–	60
Sunflower seed oil	40	40
Calcium carbonate	12	12
Dicalcium phosphate	10	10
Vitamin–mineral premix ^1^	10	10
Soybean oil	5	5
Cane molasses	5	5
Sodium chloride	2	2
Sodium bicarbonate	1	1
Metabolizable energy (kcal/kg)	2983	3264
**Chemical composition (formulated)**		
Dry matter (%)	88.66	86.51
Crude protein (%)	17.00	17.00
Ash (%)	4.81	4.80
Ether extract (%)	3.85	9.49
Crude fiber (%)	4.06	3.99

Abbreviations: LL: low lipid; HL: high lipid; DM: dry matter. ^1^ The premix contained the following nutrients (units are expressed per kg of diet): vitamin A, 15,000 IU; vitamin D3, 3000 IU; vitamin E, 25 IU; vitamin K3, 5 mg; vitamin B_1_, 2 mg; vitamin B2, 7 mg; vitamin B6, 4 mg; vitamin B12, 25 mg; pantothenic acid, 11.04 mg; nicotinic acid, 35 mg; folic acid, 1 mg; biotin, 15 μg; choline chloride, 250 mg; Cu, 1.6 mg; Mn, 60 mg; Zn, 45 mg; Fe, 80 mg; I, 0.4 mg; Se, 0.15 mg.

## Data Availability

The original contributions presented in this study are included in this article/Supplementary Materials. Further inquiries can be directed to the corresponding author.

## Supplementary Materials

The following supporting information can be downloaded at: https://www.mdpi.com/article/10.3390/vetsci13070653/s1, Figure S1: Boxplot of the expression values (normalized as TPMs) for each pooled sample. Figure S2: Principal Component Analysis (PCA) score plot of pooled samples based on whole-transcriptome expression profile. Figure S3: Hierarchical clustering heatmap of whole-transcriptome RNA-seq samples based on pairwise sample similarity. Figure S4: Gene Set Enrichment Analysis (GSEA) comparing dietary groups. Figure S5: Gene Set Enrichment Analysis (GSEA) comparing sex groups.
