# Supplementary material for: Feather RNA: A Non-Invasive Approach for Transcriptomic Profiling in Live Chickens
Source: Vet Sci. 2026 Jul 5;13(7):653. doi: 10.3390/vetsci13070653 (PMC13417430; doi:10.3390/vetsci13070653)
Supplement: Supplementary file 1 [file vetsci-13-00653-s001.zip › vetsci-4329670-supplementary.pdf]

Feather RNA: A Non-Invasive Approach for Transcriptomic Profiling in Live Chickens

Nadia Stoppani, Federica Raspa, Edoardo Fiorilla, Sandra Maione, Achille Schiavone, Cecilia Mugnai † and Dominga Soglia *,†

Dipartimento di Scienze Veterinarie, Università degli Studi di Torino, Largo Paolo Braccini 2, Grugliasco, 10095 Turin, Italy; nadia.stoppani@unito.it (N.S.); federica.raspa@unito.it (F.R.); edoardo.fiorilla@unito.it (E.F.); sandra.maione@unito.it (S.M.); achille.schiavone@unito.it (A.S.); cecilia.mugnai@unito.it (C.M.)

* Correspondence: dominga.soglia@unito.it

† These authors contributed equally to this work.

Figure S1 Boxplot of the expression values (normalized as TPMs) for each pooled sample.

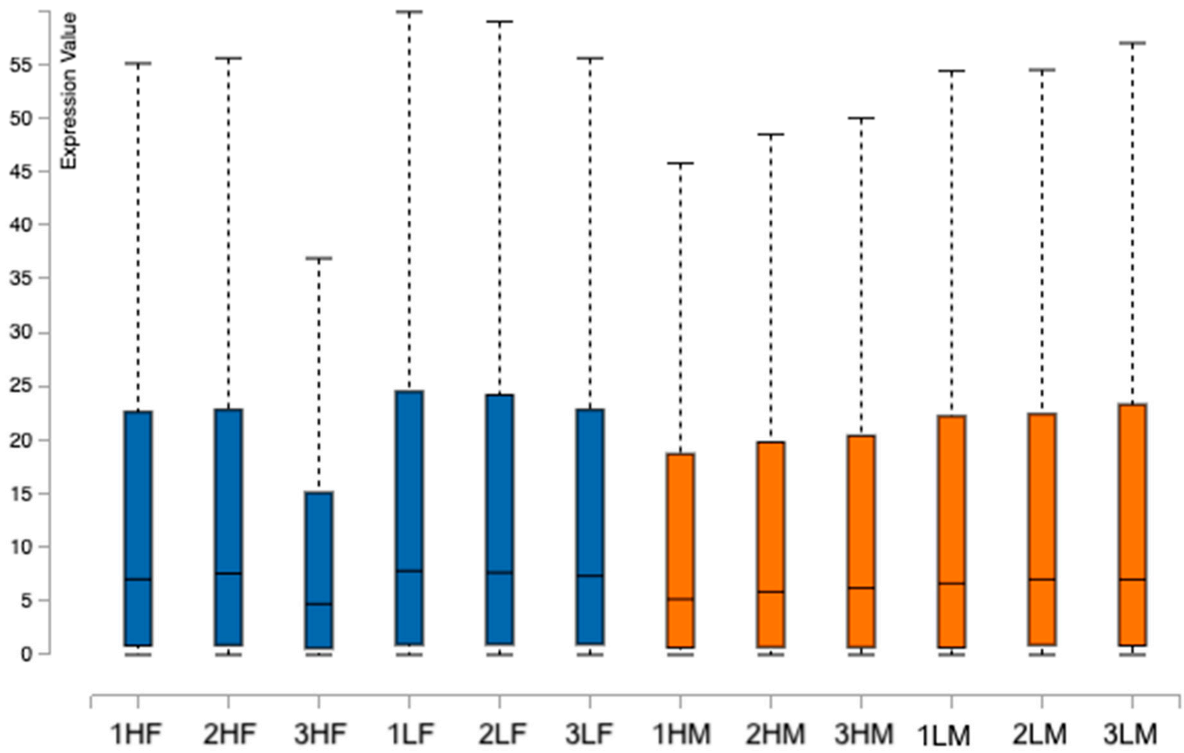

Abbreviations: HF= Females fed the high-lipidic diet; LF= Females fed the low lipidic diet; HM= Males fed the high-lipidic diet; LM= Males fed the low lipidic diet.

Figure S2 Principal Component Analysis (PCA) score plot of pooled samples based on whole-transcriptome expression profile.

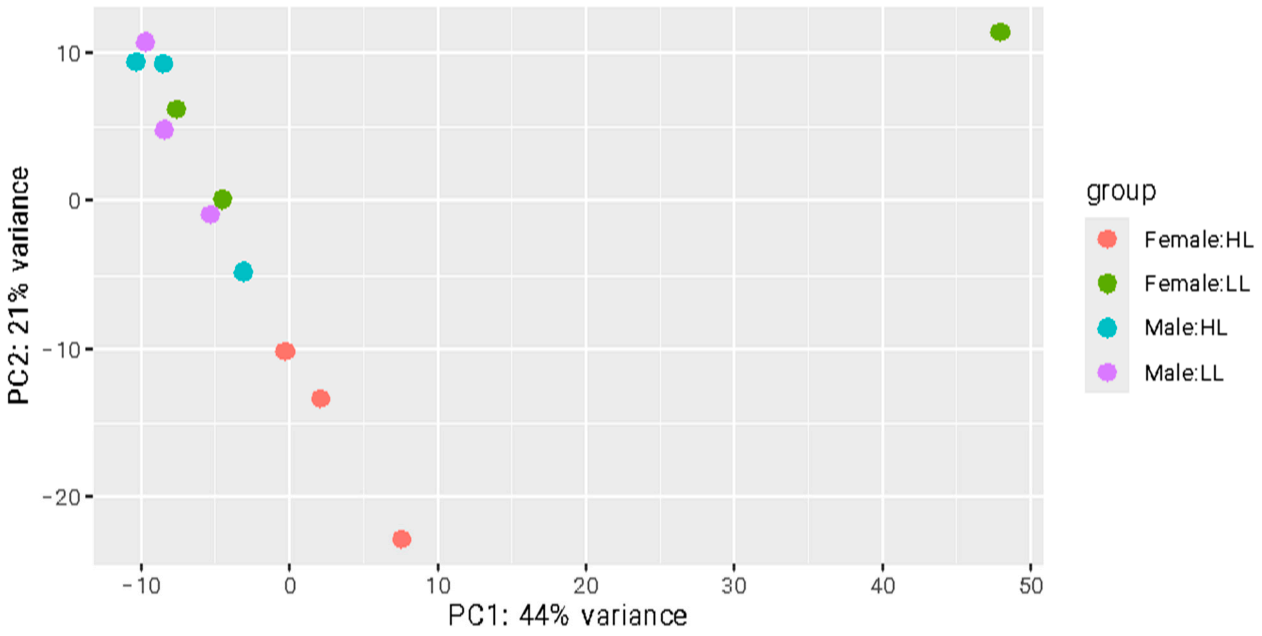

Abbreviations: HL= high-lipidic content diet; LL= low lipidic content diet

The first two principal components (PC1 and PC2) explain 44% and 21% of total variance

Figure S3 Hierarchical clustering heatmap of whole-transcriptome RNA-seq samples based on pairwise sample similarity.

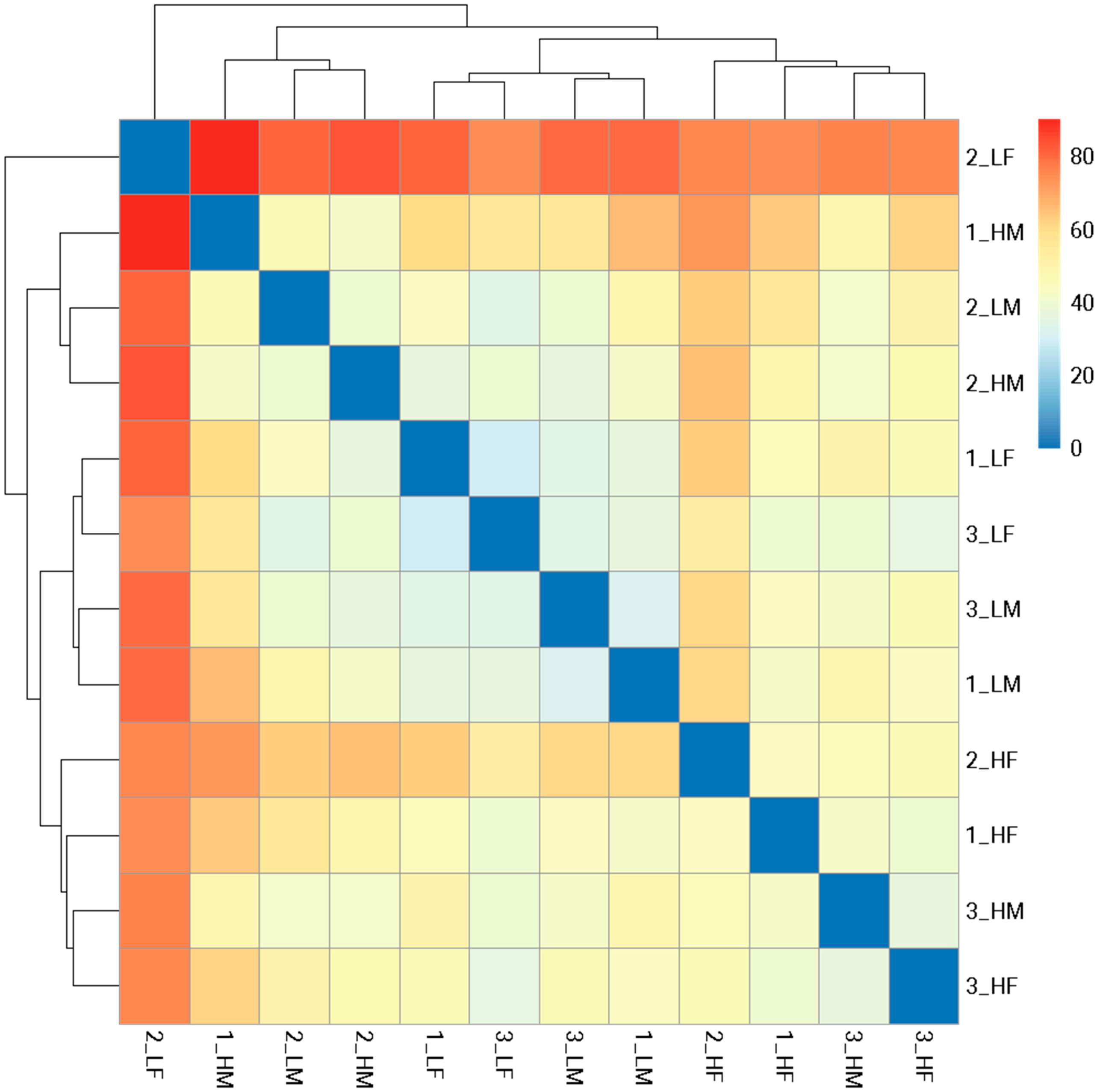

Abbreviations: HF= Females fed the high-lipidic diet; LF= Females fed the low lipidic diet; HM= Males fed the high-lipidic diet; LM= Males fed the low lipidic diet.

Color intensity represents the degree of similarity between samples, with cooler colors indicating higher similarity and warmer colors indicating lower similarity.

Figure S4 Gene Set Enrichment Analysis (GSEA) comparing dietary groups.

A)

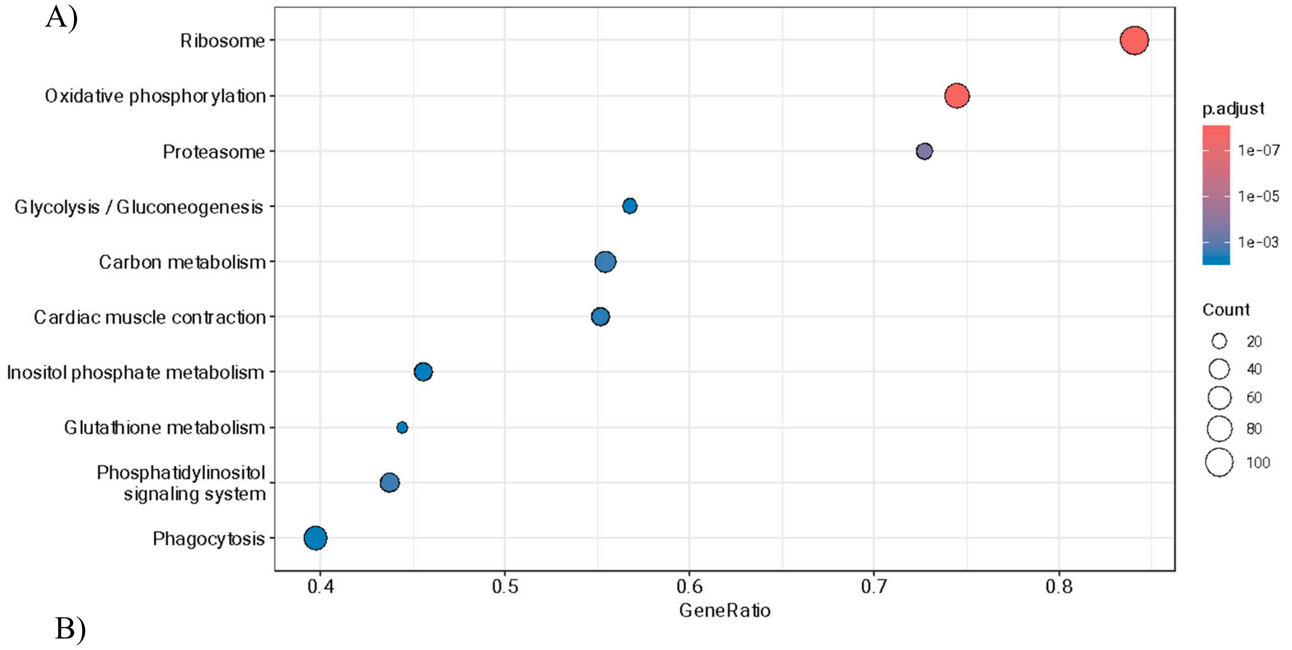

B)

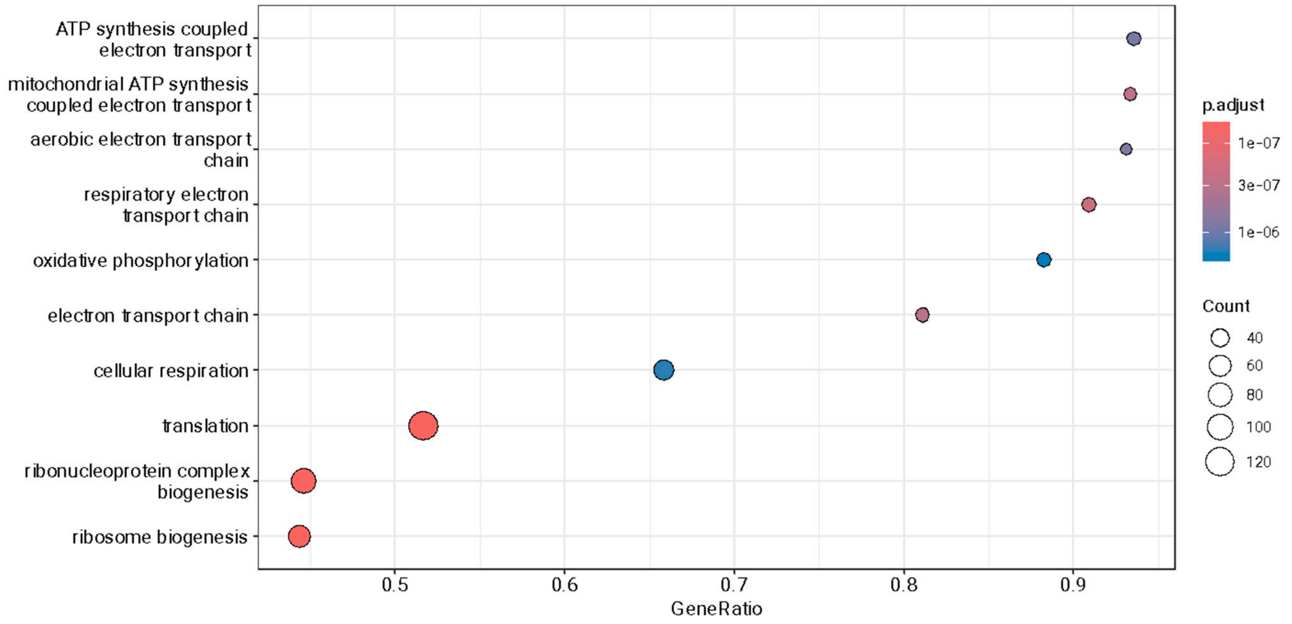

The dot plot shows significantly enriched KEGG (A) and GO (B) terms ranked by GeneRatio. Dot size represents the number of genes associated with each term, while color indicates the adjusted p-value (p-adj).

Figure S5 Gene Set Enrichment Analysis (GSEA) comparing sex groups.

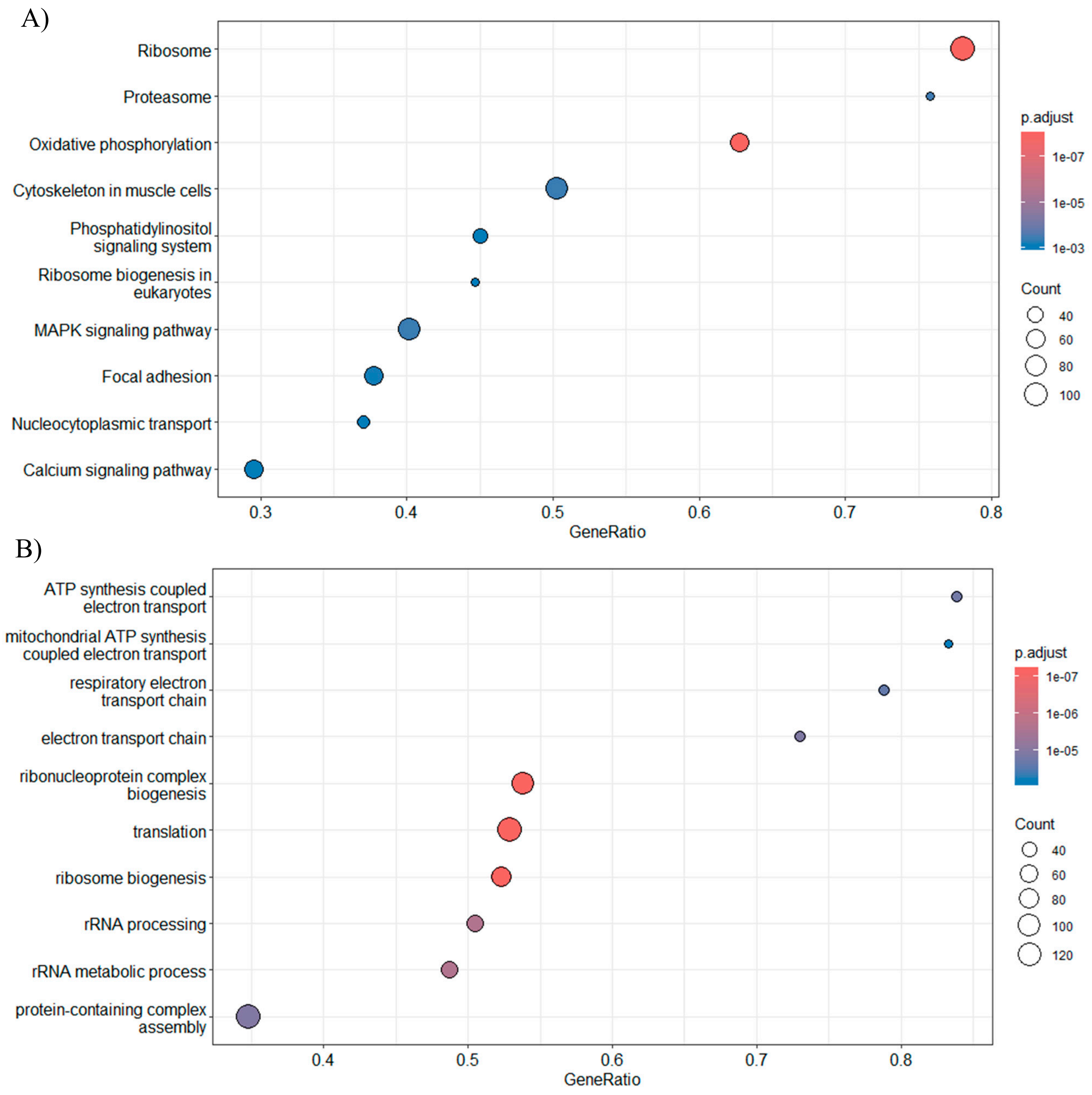

The dot plot shows significantly enriched KEGG (A) and GO (B) terms ranked by GeneRatio. Dot size represents the number of genes associated with each term, while color indicates the adjusted p-value (p-adj).
